# Pityriasis Rosea Eruption Following the Administration of Oxford-AstraZeneca Vaccine

**DOI:** 10.7759/cureus.56310

**Published:** 2024-03-17

**Authors:** Siham Marghalani, Yara Alghamdi, Bakr A Albrakati, Hassan F Huwait, Abdulrahman T Mohanna

**Affiliations:** 1 Dermatology, King Abdulaziz Medical City (KAMC), Jeddah, SAU; 2 College of Medicine, King Saud Bin Abdulaziz University for Health Sciences, Jeddah, SAU; 3 King Abdullah International Medical Research Center, Ministry of the National Guard - Health Affairs, Jeddah, SAU; 4 Dermatology, Hera General Hospital, Makkah, SAU; 5 Dermatopathology, Umm Al-Qura University, Jeddah, SAU

**Keywords:** drug-related side effects and adverse reactions, pfizer-biontech bnt162b2, pfizer-biontech covid-19 vaccine, astra zeneca covid-19 vaccine, oxford-astrazeneca vaccine, pityriasis rosea

## Abstract

The coronavirus disease 2019 (COVID-19) infection has led to accelerated development and utilization of vaccines to prevent its implications on health. One of these vaccines is a vector-based, Oxford-AstraZeneca Vaccine (AZD1222). Frequently reported side effects are related to host-immune response. While dermatologic manifestation is peculiar in nature and denotes a serious eruption that might defer future vaccination. Herein, we present a case of a medically free 37-year-old female who developed clinical and histological evidence of pityriasis rosea (PR) after administration of a second-dose vaccination of AZD1222. The first dose of vaccination was administered as Pfizer BioNTech COVID-19 mRNA (BNT162b2) vaccine. This case is unique in nature as this patient developed AZD1222-induced PR, while some reports in the literature have linked PR to the BNT162b2 vaccine. This patient continued to receive a booster vaccination with BNT162b2 with no reportable side effects.

## Introduction

The novel coronavirus disease 2019 (nCOVID-19) caused a health calamity that led to the rapid development of vaccines to prevent severe acute respiratory virus coronavirus 2 (SARS-CoV-2) transmission and hospitalization. There is an element of rapid acceleration in the process of vaccine approval which raises the issue of safety concerns. To this day, vaccines against nCOVID-19 have undergone emergency use authorization (EUA) to expedite the process of administration to the public. One of those vaccines is Pfizer BioNTech COVID-19 mRNA (BNT162b2). This vaccine was approved for EUA in the United States (US) and is 95% efficient in decreasing symptomatic SARS-CoV-2 infection in persons who lack evidence of previous nCOVID-19 infection [1]. Frequently reported side effects are fatigue, headache, muscular pain, chills, and injection site pain [1]. Cutaneous manifestations are uncommon and, if present, they are of a self-limited nature and should not discourage the utilization of the vaccine [2]. Cutaneous eruptions that have been reported among BNT162b2 vaccine recipients are urticarial rash, local injection site pain, and morbilliform (measle-like) rash [2]. McMahon performed a case-based registry in the US and reported the characteristics of frequently encountered cutaneous eruptions that were noticed after second dose administration, specifically, delayed large local reactions, followed by local injection reaction, urticarial rash, morbilliform manifestations, and papulosquamous eruptions [2,3]. Another vaccine that proved to be efficacious against nCOVID-19 infection is the Oxford-AstraZeneca COVID-19 vaccine (AZD1222) [4]. This vaccine was approved through EUA in the United Kingdom (UK) and is given in two doses within a 4-12-week interval [4]. The commonly reported adverse reactions are injection site pain, fever, headache, nausea, and vomiting [5]. Cutaneous eruptions following the AZD1222 vaccine were also reported, and the frequently encountered manifestation was urticaria [5,6]. AZD1222 trial analyzed that local injection site reactions and cases of psoriasis, rosacea, vitiligo, and Raynaud's syndrome have been reported amongst vaccine recipients [3]. This report is of a 37-year-old healthy female who developed generalized pruritic erythematous fine scaly eruptions on the trunk and proximal extremities that materialized after vaccinating with AZD1222 and from clinical and histological perspectives, a diagnosis of pityriasis rosea (PR) eruption was established.

## Case presentation

A medically free 37-year-old female presented with pruritic hyperkeratotic erythematous scaly eruptions on the trunk and proximal extremities that manifested after administration of the AZD1222 vaccine. Cutaneous manifestations materialized two days post-vaccination, along with 39.5 degrees Celsius fever and arm heaviness. The eruption started around the breasts as pruritic erythematous papules that progressed into well-demarcated regular erythematous to salmon-colored plaques with scales on the periphery, and then migrated to the trunk and proximal thighs following Langer’s line of cleavage.

After five days, the eruption progressed to be generalized and a Christmas tree pattern could be observed over the trunk. Fever and arm-heaviness persisted for two weeks post-vaccination, while cutaneous eruption which was thought to be of fungal origin had persisted for four weeks with no improvement with topical emollient, mometasone furoate 0.1% ointment, and miconazole cream.

**Figure 1:**
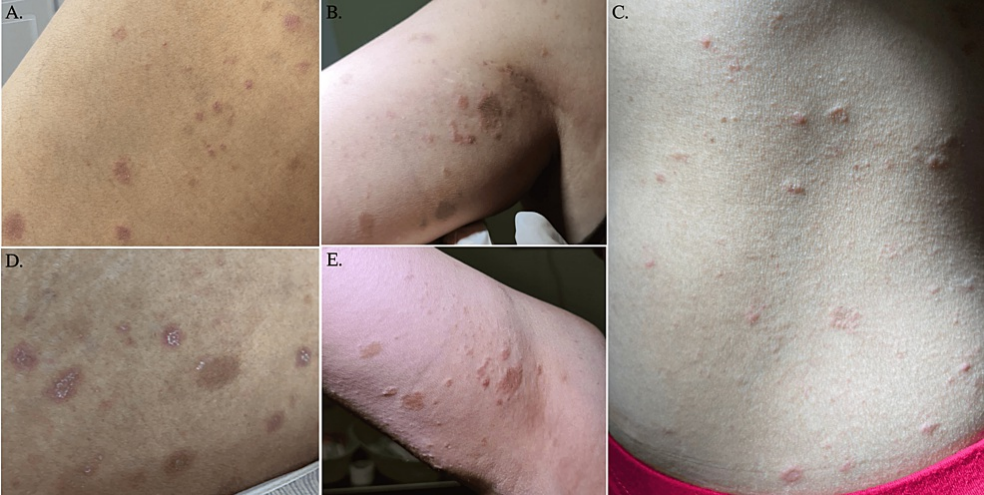
PR after administration of AZD1222 vaccine Oval-shaped, erythematous to salmon-colored papules and plaques with a peripheral collarette of scales over the proximal right thigh (A), medial right upper arm (B), and across the abdomen in a Christmas tree pattern (C). Oval erythematous-violaceous papules and plaques with a central fine white scale formation and signs of excoriation over the lower abdomen (D), erythematous papules with small fine scales following skin’s cleavage lines over proximal right arm (E). PR: pityriasis rosea

After two weeks, the patient presented to the dermatology office with worsening pruritis and erythematous to salmon-colored fine scaly papules and plaques (shown in Figure 1). The herald patch was on the breast, but the patient only consented to photography of lesions on the trunk and extremities.

**Figure 2:**
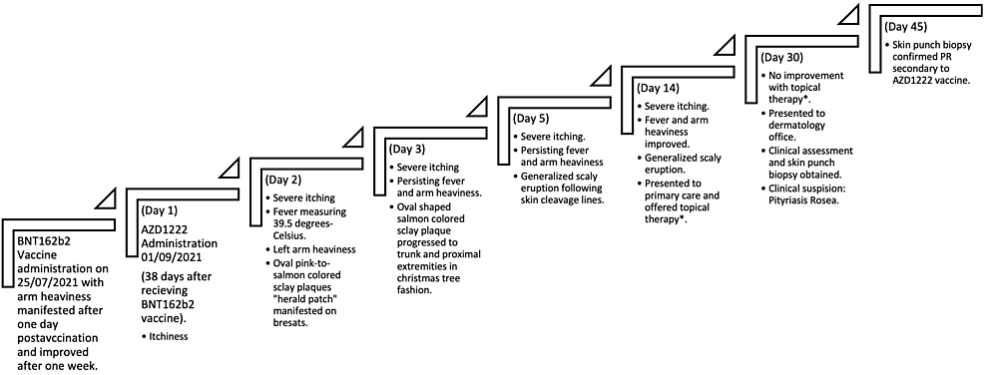
The temporal manifestation of PR eruption on day 2 after receiving the AZD1222 vaccine PR: pityriasis rosea

There was no mucous membrane, axillae, or groin involvement. No history of human herpesviruses (HHV-6 and HHV-7) infection. No history of prodromal illness, known allergies, or previous history of similar lesions erupting. The patient had previously received the BNT162b2 vaccine 38 days before receiving the AZD1222 vaccine, and merely arm heaviness was experienced (timeline is shown in Figure 2).

**Figure 3:**
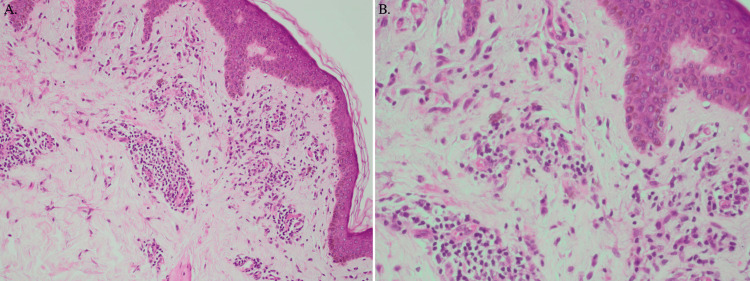
Histopathology of PR (A) Hematoxylin and Eosin (H&E)-stained section shows superficial and mid-dermal perivascular lymphocytic infiltrate (H&E x100). (B) The H&E-stained section shows focal RBC extravasation. Overall, features are suggestive of PR (H&E x200). PR: pityriasis rosea

From a clinical perspective, PR was suspected and, later, confirmed by skin punch biopsy. Histopathological sections revealed orthokeratotic stratum corneum, unremarkable epidermis, and superficial perivascular lymphocytic infiltrate with red blood cell extravasation and pigment incontinence within the dermis, consistent with PR (shown in Figure 3).

From clinical and histopathological evaluation, PR eruption secondary to AZD1222 vaccine was established. The patient was managed with prednisolone 5mg/day for seven days, topical betamethasone valerate 0.1% cream, and topical pimecrolimus 1% cream. On a follow-up examination after one month, an improvement of cutaneous lesions was observed with post-inflammatory hyperpigmentation evident on previously active lesions.

A booster vaccine with BNT162b2 had been administered without any reported side effects. There is no clear contraindication for receiving any future doses of the nCOVID-19 vaccine. Nonetheless, vector-based vaccines must be avoided and immunity with vaccination might be achieved with mRNA-based vaccines as the first dose vaccination with the BNT162b2 vaccine did not illicit any dermatological manifestation. The patient is following up routinely with our dermatology clinic in case another eruption occurred, or a PR flare-up is manifested.

## Discussion

The EUA of both BNT162b2 and AZD1222 vaccines after ensuring efficacy, absence of serious adverse events, and tolerable side effects deemed them safe for the public [1-5]. BNT162b2 is an mRNA-based vaccine that encodes the SARS-CoV-2 spike protein in a lipid nanoparticle [5]. However, AZD1222 is a recombinant adenovirus vector-based vaccine from chimpanzees, and it encodes SARS-CoV-2 spike-glycoprotein [5]. The two vaccines are of different modes of delivery and mechanisms of immunogenicity. Nonetheless, both were deemed effective in preventing SARS-CoV-2 infection in seronegative recipients. The literature is lacking in immunogenicity data that reports a mixture of the two vaccines could lead to side effects, specifically, cutaneous manifestations. The patient in this report developed PR after receiving AZD1222 (shown in Figure 2), her sister received the same mixture of the two vaccines, BNT162b2 and AZD1222, without developing any cutaneous manifestation.

PR is a papulosquamous manifestation of acute onset and a self-limited nature [7]. The eruption follows the skin cleavage lines forming a pattern like a Christmas tree. PR frequently affects adolescents and young adults in a self-limited manner [7,8]. Pathophysiology is unknown, but it could be linked to viral illnesses [7]. Vaccination against nCOVID-19 could lead to a PR-like eruption. There is a magnitude of reports on PR eruption following nCOVID-19 vaccination [8-12]. The commonly implicated vaccine that was vigorously reported in the literature is an mRNA vaccine, namely BNT162b2 [8-11]. The PR-like eruption following COVID-19 vaccination presents typically on the site of administration as an erythematous plaque with outer fine whitish scaling, and later, the cutaneous manifestation progresses to be generalized following the skin cleavage lines [7-12]. This manifestation typically presents after the second dose administration and affects females according to reported cases from Turkey, Lebanon, Canada, Spain, and Germany [7-12]. There were only three reports implicating the AZD1222 vaccine in PR eruption (shown in Table 1) [5,13,14]. Two cases of females aged 52 and 53 years developed PR two weeks after receiving AZD1222. No prodromal symptoms were experienced. Management was successful with topical treatment [5,13]. While a male aged 24 developed PR two days after receiving the AZD1222 vaccine. Lesions subsided with topical therapy [14]. All reported cases were similar to this case in the manner of widespread PR eruption.

**Table 1:** PR secondary to AZD1222 reported cases ^1^ Diagnosis of PR post-vaccination with AZD1222 was established on clinical grounds by a dermatologist and infectious disease specialist. ^2^ First dose: AZD1222 and eight hours post-vaccination a 9-degree fever, joint pain, chills, and headache developed. ^3^ Diagnosis was based on clinical presentation and supported by dermatoscopic findings of the irregular collection of fine white scales, patchy distribution of dotted vessels on the periphery, and brown globules over a diffuse reddish-brown background. PR: pityriasis rosea

References	Age (years)	Gender	Nationality	Co-morbidities	Prodromal illness	Presentation	Histopathology (if reported)	Vaccine, dose	Management
Leerunyakul K et al. [13]	52	Female	Thai	Glioblastoma on levetiracetam and thyroxin	No fever or respiratory tract symptoms	Fourteen days after vaccination, multiple blanching erythematous oval patches and plaques on the lower abdomen, back, and bilateral upper thighs. No herald patch or scale.	Epidermal focal spongiosis, interface dermatitis with necrotic keratinocytes, perivascular lymphocytic infiltration, and eosinophilic infiltrates in the superficial dermis	AZD1222, first dose	Topical 0.1% triamcinolone acetonide twice daily with moderate response after seven days.
Pedrazini M et al. [5]	53	Female	Brazilian	Hashimoto's Thyroiditis on 125 mg levothyroxine sodium	Not reported	Fifteen days after vaccination, erythematous plaques with mild itching on the posterior right thigh and above knee joint. Then progressed to calf and buttocks. Herald patch on the right thigh and posterior knee.	Not reported^1^	AZD1222, second dose^2^	1.5 mg/g bismuth subgallate gall and zinc oxide 45.0 mg/g. L-Lysine 3 grams single dose, followed by 500 mg/day for 30 days showed no improvement. The eruption subsided in the eighth week.
Mehta H [14]	24	Male	Indian	Not Reported	NA	Twenty-four hours after vaccination, multiple well-defined round-to-oval salmon-colored papules and plaques measuring 3 cm and covered by fine white scales over the trunk, back, and axillae.	Did not consent for biopsy^3^	AZD1222, first dose	Topical mometasone cream. Progression of PR eruption could not be monitored as the patient lost to follow-up.

Due to the newness of the nCOVID-19 vaccines, there is an importance in reporting side effects, namely cutaneous eruptions. There are no reports from the literature on similar circumstances that lead to cutaneous eruptions similar to the case mentioned above. To our knowledge, this is the first case in literature that reports on PR eruption after the AZD1222 vaccine with a previously administered vaccine is BNT162b2. There are recent reports that highlight BNT162b2 vaccines could elicit PR-like eruption [7-12]. Although these eruptions are harmless and self-limiting in nature, they cause anxiety to the patient from a cosmetic perspective due to post-inflammatory hyperpigmentation. More data and future recommendations should be developed to predict such eruptions in susceptible patients to decrease the cutaneous manifestation burden. Nonetheless, these eruptions should not discourage vaccination.

## Conclusions

There is a huge question mark in regard to the safety of COVID-19 vaccines. Nonetheless, vaccination outweighs the risk of infection and poor outcomes. This report delineates the unusual development of PR with AZD1222, although the first dose vaccination was with BNT162b2. A booster dose was administered with BNT162b2 without any reported side effects. Reports circling around the mixture of the two vaccines are lacking; thus, this paper will add great insight into future mRNA-based and vector-based vaccines and their possible cutaneous manifestations.
